# What’s important to you? Socioeconomic inequalities in the perceived importance of health compared to other life domains

**DOI:** 10.1186/s12889-022-12508-2

**Published:** 2022-01-13

**Authors:** Sanne E. Verra, Maartje P. Poelman, Andrea L. Mudd, Emely de Vet, Sofie van Rongen, John de Wit, Carlijn B.M. Kamphuis

**Affiliations:** 1 Department of Interdisciplinary Social Science, Utrecht University, Padualaan 14, 3584 CH Utrecht, the Netherlands; 2 Chair group Consumption and Healthy Lifestyles, Wageningen University & Research, Hollandseweg 1, 6706 KN Wageningen, the Netherlands

## Abstract

**Background:**

Pressing issues, like financial concerns, may outweigh the importance people attach to health. This study tested whether health, compared to other life domains, was considered more important by people in high versus low socioeconomic positions, with future focus and financial strain as potential explanatory factors.

**Methods:**

A cross-sectional survey was conducted in 2019 among N=1,330 Dutch adults. Participants rated the importance of two health-related domains (not being ill, living a long life) and seven other life domains (e.g., work, family) on a five-point scale. A latent class analysis grouped participants in classes with similar patterns of importance ratings. Differences in class membership according to socioeconomic position (indicated by income and education) were examined using structural equation modelling, with future focus and financial strain as mediators.

**Results:**

Three classes were identified, which were defined as: *neutralists*, who found all domains neutral or unimportant (3.5% of the sample); *hedonists*, who found most domains important except living a long life, work, and religion (36.2%); and *maximalists*, who found nearly all domains important, including both health domains (60.3%). Of the *neutralists*, 38% considered not being ill important, and 30% considered living a long life important. For *hedonists*, this was 92% and 39%, respectively, and for *maximalists* this was 99% and 87%, respectively. Compared to belonging to the *maximalists* class, a low income predicted belonging to the *neutralists*, and a higher educational level and unemployment predicted belonging to the *hedonists*. No mediation pathways via future focus or financial strain were found.

**Conclusions:**

Lower income groups were less likely to consider not being ill important. Those without paid employment and those with a higher educational level were less likely to consider living a long life important. Neither future focus nor financial strain explained these inequalities. Future research should investigate socioeconomic differences in conceptualisations of health, and if inequalities in the perceived importance of health are associated with inequalities in health. To support individuals dealing with challenging circumstances in daily life, health-promoting interventions could align to the life domains perceived important to reach their target group and to prevent widening socioeconomic health inequalities.

**Supplementary Information:**

The online version contains supplementary material available at 10.1186/s12889-022-12508-2.

## Background

Health inequalities are widening, even in countries with elaborate welfare systems [1]. Those with a lower socioeconomic position (SEP) often face unfavourable material and psychosocial conditions [2, 3]. The accumulation of material and psychosocial risk factors contributes to large inequalities in health. In the Netherlands, this translates to an average gap of eighteen healthy life years between those with a lower and higher SEP [4]. Alongside material and environmental factors, a part of these health inequalities results from differences in health behaviour [1, 5, 6].

A potential explanation for socioeconomic inequalities in health is that taking care of one’s health may be less prominent in the lives of those with a lower SEP compared to those with a higher SEP [7]. Health is often considered a universal value [8]. Yet, several studies showed that when comparing the importance of health to other life domains, it is not given equal weight by everyone [9–12]. Schneider and Barnes (2003) analysed the importance of different life goals as motivators for decision-making and found that health was generally identified as less important than other goals, such as relationships and careers [10]. In daily life, health competes with many other priorities, such as work and social obligations. Other priorities frequently outweigh the perceived importance of health [9].

One reason it may be difficult to prioritise health over other life domains is that health behaviours mainly pay off in the future, whereas investments in other life domains, such as spending time with friends, result in more immediate benefits. Studies have shown that those with a lower SEP tend to focus more on the present than those with a higher SEP [13, 14]. Having a future focused orientation instead of a focus on the present has been linked to healthier dietary behaviour [15] and could mediate socioeconomic inequalities in the perceived importance of health.

Financial strain is another potential mediator of the relationship between SEP and the importance of health. Scarcity theory posits that the stress to make ends meet may take up a lot of cognitive capacity, leaving little capacity to deal with less urgent matters [16–18]. Although an increased focus on pressing issues like financial strain helps in dealing with the current situation, a less urgent and more long-term goal such as staying healthy may receive less attention [16]. As a result, the perceived importance of health compared to other life domains could be lower among those with a lower SEP compared to those with a higher SEP, as those with a lower SEP are more likely to experience financial strain [19].

Inequalities in the perceived importance of health compared to other life domains are understudied [8, 9, 20]. We hypothesize that people with a lower SEP are more likely than people with a higher SEP to perceive health as less important compared to other life domains (such as financial situation, work or leisure time). Explanations may be that people with a lower SEP are less likely to have a future focused orientation, or that challenging issues in other life domains could outweigh the perceived importance of health. The aims of this study are: (1) to identify if and to what extent there are socioeconomic inequalities in the perceived importance of health compared to other life domains, and (2) to what extent these socioeconomic inequalities in the perceived importance of health are mediated by future focus and financial strain.

## Methods

### Design and study population

A cross-sectional survey was undertaken in January 2019. Participants were recruited from a panel established by an online research agency [21]. Individuals between 25 and 60 years old who were not enrolled in education could participate. The survey was completed by 1,336 participants (59% response rate, mean age = 44.8, SD = 10.4, 57% female, 95% identified as Dutch). Lower income panel members were oversampled to compensate for their potential relatively lower response rate [22, 23]. This led to the inclusion of 531 low, 404 middle, and 401 high income participants. Two cases with missing values for gender were excluded, as well as four cases with a missing value for educational level, resulting in an analytical sample of N=1,330. The sample was representative of the Dutch population with regards to gender, age, educational level, and province but not income [24]. Compared to the income distribution in the Dutch population, lower incomes were overrepresented in our sample [25]. Ethical approval was obtained from the ethics committee of the faculty of Science and Geo Sciences of Utrecht University (GEO FETC18-014).

### Measures

#### Socioeconomic position

Income and education were used as separate indicators of SEP, as both measure different aspects of SEP [26]. The online research agency supplied data on gross household income of participating panel members, categorised as low (<€13.300 per year), middle (€13.300 - €41.200), and high (>€41.200 per year). Participants reported their highest completed level of education in the survey. Following ISCED 2011 classifications, education was categorised as low (lower secondary education at most; ISCED 0-2), middle (upper secondary education; ISCED 3-4), or high (tertiary education; ISCED 5-6).

#### Importance of health compared to other life domains

Based on the approach by Hsieh, [11] participants rated the importance of nine items representing life domains on a five-point Likert scale (1 = “very unimportant”, 5 = “very important”): work, spare time, financial situation, neighbourhood, family life, friendships, religion, and two health domains. We included two health domains, since a Dutch qualitative study [27] found those with a lower education more likely to conceptualise health in mostly negative terms (e.g., “absence of disease”), while the more highly educated also included more positive aspects, such as “lust for life” and “vitality”. To account for possible differences in the conceptualisation of health, the importance of two distinct health-related items was assessed: “not being ill” (a negative frame) and “living a long life” (a positive frame). For all nine items, the five-point Likert scale answers were dichotomised (“very unimportant”, “unimportant”, and “neutral” categorized as unimportant, and “important” and “very important” categorized as important) to improve interpretability of the latent class analysis (LCA) results (explained under statistical analysis).

#### Future focus

Future focus was measured using the Temporal Focus Scale [28]. Four future-focused items, for example, “My mind is on the here and now” and “I think about what my future has in store” (Cronbach’s α = 0.86), were included. Participants answered on a five-point scale (1 = “never”, 5 = “constantly”). Based on the average scores across items, three categories were created: low (mean score ≤ 2), middle (> 2 and ≤ 3), and high (> 3) future orientation.

#### Financial strain

Financial strain was assessed with six items (Cronbach’s α = 0.95). To create a combined financial strain measure, the answer categories of each financial strain item were divided into low, medium, and high financial strain. Two items asked about financial strain in the preceding year: “Have you experienced difficulty paying for your food, rent, and bills from your household income?” with responses considered as low (“no difficulty”), medium (“some difficulty”), or high (“a lot of difficulty”), and “How is your household getting by?” with four response options, considered as low (“easily”, “somewhat easily”), medium (“with some difficulty”), and high (“with large difficulty”). Four items asked about daily financial strain, for example: “How often in the last four weeks did you worry about your financial situation?”, measured on a five-point scale. Responses were considered as low (“never” and “rarely”), medium (“sometimes”), and high (“often” and “constantly”). Participants were assigned a low, medium or high financial strain ranking based on their highest answer (low, medium or high) across the six items.

#### Potential confounders

Age, gender, and paid employment were included as confounders, as previous research pointed to their influence in the perceived importance of life domains, [10, 12, 29] and they have been associated with SEP. Age was included as a continuous variable and gender as a binary variable (1 = female, 0 = male). Paid employment was assessed as a binary variable (1 = in paid employment, 0 = not in paid employment). Paid employment status was not used as an indicator of SEP since it does not capture what occupational class the person is employed in [30].

### Statistical analysis

This study assessed the perceived importance rating of the different life domains using a LCA. The outcome of the LCA, or ”class membership”, was used as dependent variable in subsequent models.

The LCA distributed participants in classes based on patterns in their importance ratings of the nine life domains. Different LCA models were tested, predicting two to six classes. To find the amount of classes with the highest likelihood, 5,000 iterations were run with 30 different starting values for each model. To choose the optimal number of classes, we drew on the Bayesian Information Criterion (BIC), a model fit measure that penalises model complexity more than the Akaike Information Criterion (AIC) [31]. No data were missing in this part of the analysis. Each participant was assigned to the class for which their membership probability was largest [32, 33]. The LCA was conducted in R, using poLCA version 1.4.1 [34].

A structural equation model (SEM) was built to examine whether future focus and financial strain mediated the relationship between SEP and class membership. The approach used by Nguyen et al. [35] was followed, as this allowed for multiple ordinal mediators and binary outcomes. Predictors included income and educational level as indicators of SEP, future focus and financial strain as mediators, and age, gender and employment as confounders. The step-by-step procedure from Zhao et al. was used to test for mediation [36]. The SEM with probit link used a Weighted Least Square Mean and Variance adjusted estimator and was run in Mplus version 8.4 [37].

## Results

### Latent Class Analysis

Model fit statistics of the LCA showed that the three-class model had the most optimal BIC value (see Table 1). This model allowed for the classification of participants into three meaningful classes: (1) *neutralists*, who were likely to rate all domains neutral or unimportant (3.5% of the sample, n=47), (2) *hedonists*, who were likely to find many domains important, except for living a long life, work, and religion (36.2%, n=482), and (3) *maximalists*, who were likely to find all life domains important, except religion (60.3%, N=802). Note that the names used to describe the three classes were based on the researchers’ interpretation of the characteristics of the mentioned classes.

**Table 1:** Key statistics for different LCA models

Classes	Log- likelihood	Degrees of freedom	BIC	AIC
2	-4,925.314	492	9,987.295	9,888.629
3	-4,842.475	482	9,893.545	9,742.950
4	-4,822.027	472	9,924.578	9.722.053
5	-4,808.963	462	9,970.380	9,715.926
6	-4,793.674	452	10,011.73	9,705.348

**Fig. 1:**
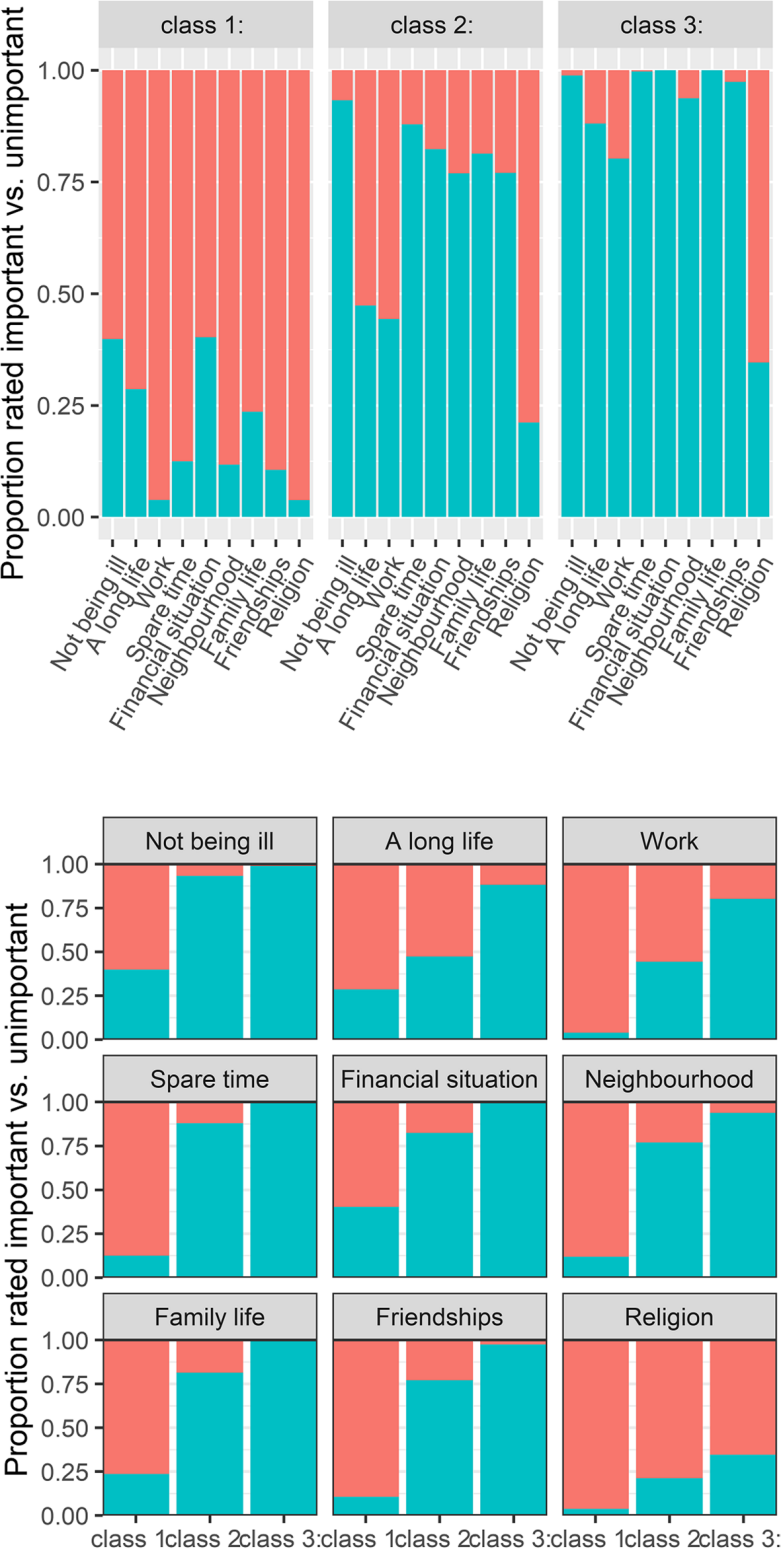
The proportion of life domains considered important vs. unimportant per class (above) and per life domain (below). Class 1: neutralists, class 2: hedonists, class 3: maximalists

Figure 1; Table 2 depict how health and other life domains were rated by the different classes. Overall, those in the *neutralists* class rated all domains neutrally important, including both health domains. The domains that *neutralists* most often rated important were not being ill (rated important by 38%, see Table 2), financial situation (rated important by 34%), and family (rated important by 21%). Least important to *neutralists* were religion (rated important by 4%), work (rated important by 4%), and friendships (rated important by 11%).

**Table 2:** Sample characteristics by class membership

		Total N=1,330	*Neutralists* (n=47)	*Hedonists* *(*n=482)	*Maximalists* (n=801)	*Χ*^2^ *statistic*^a^
**Education**	Low	27%	45%	26%	26%	11.11
	Middle	46%	40%	48%	45%	*
	High	27%	15%	26%	29%	
**Yearly**	<€13,300	40%	79%	45%	34%	58.72
**household**	€13,300-41,200	30%	19%	32%	30%	***
**income**	>€41,200	30%	2%	23%	36%	
**Age**	25-35	22%	13%	21%	23%	4.67
	35-45	24%	28%	23%	24%	ns
	45-55	32%	40%	32%	32%	
	55-60	23%	19%	24%	22%	
**Gender**	Female	57%	68%	56%	58%	2.47
	Male	43%	32%	44%	42%	ns
**Employment**	Paid	63%	36%	52%	71%	62.36
**status**	Other	37%	64%	48%	29%	***
**Ethnicity**	Dutch	95%	96%	95%	95%	0.32
	Non-Dutch	5%	4%	5%	5%	ns
**Future focus**	Low	4%	11%	6%	3%	39.18
	Middle	35%	47%	42%	31%	***
	High	61%	42%	52%	66%	
**Financial**	Low	18%	8%	17%	20%	16.02
**strain**	Middle	33%	28%	29%	35%	**
	High	49%	64%	54%	45%	
**Not being ill**	Important	94%	38%	92%	99%	308.66
	Unimportant	6%	62%	8%	1%	***
**Living a long**	Important	68%	30%	39%	87%	341.93
**life**	Unimportant	32%	70%	61%	13%	***
**Spare time**	Important	91%	11%	85%	100%	487.06 ***
	Unimportant	9%	89%	15%	0%	
**Financial situation**	Important	90%	34%	79%	100%	319.60 ***
	Unimportant	10%	66%	21%	0%	
**Family**	Important	89%	21%	77%	100%	386.48 ***
	Unimportant	11%	79%	23%	0%	
**Friendships**	Important	85%	11%	68%	100%	456.96 ***
	Unimportant	15%	89%	32%	0%	
**Neighbour-hood**	Important	83%	9%	73%	94%	293.62 ***
	Unimportant	17%	91%	27%	6%	
**Work**	Important	61%	4%	37%	79%	288.81 ***
	Unimportant	39%	96%	63%	21%	
**Religion**	Important	27%	4%	23%	32%	24.76 ***
	Unimportant	73%	96%	77%	68%	

^a^Significance levels are reported based on * *p*<0.05, ** *p*<0.01, *** *p*<0.001

Those in the *hedonists* class were more likely to rate life domains important than those in the *neutralists* class. The domains that *hedonists* rated most often important were not being ill (rated important by 92%), spare time (rated important by 85%), and financial situation (rated important by 79%). Least important to *hedonists* were religion (rated important by 23%), work (rated important by 37%), and living a long life (rated important by 39%).

Those in the *maximalists* class were more likely to rate life domains important than those in the *neutralists* and *hedonists* classes. The domains *maximalists* rated most often important were family (rated important by 100%), spare time (rated important by 100%), and not being ill (rated important by 99%). Least important to *maximalists* were religion (rated important by 32%), work (rated important by 79%), and living a long life (rated important by 87%).

All classes rated not being ill as most important compared to the other life domains, yet the probability of perceiving not being ill important was lower for *neutralists* and *hedonists* compared to *maximalists.* The *hedonists* and the *maximalists* considered living a long life as one of the least important life domains. Relatively, living a long life was considered similarly important as the other life domains for those in the *maximalists* and *neutralists* classes, but living a long life was considered substantially less important than other life domains among those in the *hedonists* class.

Table 2 shows the demographic characteristics of participants in each class. Compared to the other classes, those in the *neutralists* class were most likely to have a low educational level, low income level, no paid employment, to be less future focused, and to experience high financial strain. Those in the *hedonists* class were most likely to have a middle educational level, and to belong to the middle income category. Those in the *hedonists* class were also less likely to have paid employment, to be future focused, and more likely to experience financial strain compared to those in the *maximalists* class.

SEMs were built for two outcome variables: predicting the probability of belonging to the *neutralists* class compared to the *maximalists* class and predicting the probability of belonging to the *hedonists* class compared to the *maximalists* class. As those in the *maximalists* class had the highest overall domain ratings, this was used as reference class. The SEMs were built in three steps, by including (1) income and education; (2) confounders, and (3) mediators. This resulted in a total of six SEMs. Table 3 presents the main model results. Note that only the effects for class membership and mediators as dependent variables are presented. More effects (such as the influence of the confounders on our SEP variables, and the relationship between our SEP variables) were estimated in the models, but excluded from Table 3. See Supplementary File 1 for a graphical representation of all relationships estimated in model 3.

**Table 3:** Results of structural equation models

	Outcome	Predictors	*Neutralists* vs. *Maximalists*^a^	*Hedonists* vs. *Maximalists*
Model 1: Income and educational level	Class membership	Income	-0.68(0.14)***	-0.23(0.05)***
		Education	-0.09(0.11)	0.05(0.05)
	**CFI**^**b**^		1.00	1.00
Model 2: Model 1 + confounders	Class membership	Income	-0.47(0.11)***	-0.11(0.04)*
		Education	0.01(0.10)	0.09(0.05)
		Employment	-0.15(0.20)	-0.41(0.10)***
		Age	-0.00(0.01)	0.00(0.01)
		Gender	-0.20(0.18)	-0.19(0.08)*
	**CFI**		0.89	0.89
Model 3: Model 2 + mediators	Class membership	Income	-0.52(0.14)***	-0.09(0.05)
		Education	0.00(0.11)	0.10(0.05)*
		Financial strain	-0.08(0.11)	0.03(0.05)
		Future focus	-0.30(0.09)**	-0.22(0.05)***
		Employment	-0.10(0.20)	-0.37(0.10)***
		Age	-0.01(0.01)	-0.00(0.00)
		Gender	-0.16(0.19)	-0.17(0.08)*
	Future focus	Income	0.02(0.04)	0.02(0.04)
		Education	0.03(0.04)	0.03(0.04)
		Employment	0.20(0.09)*	0.20(0.09)*
		Age	-0.02(0.00)***	-0.02(0.00)***
		Gender	0.15(0.07)*	0.15(0.07)*
	Financial strain	Income	-0.49(0.05)***	-0.49(0.05)***
		Education	-0.03(0.04)	-0.03(0.04)
		Employment	-0.10(0.09)	-0.11(0.09)
		Age	0.00(0.00)	0.00(0.00)
		Gender	0.07(0.07)	0.07(0.07)
	Financial strain with	Future focus	0.07(0.04)	0.07(0.04)
	**CFI**		0.92	0.92

^a^Probit coefficients, standard errors in parentheses, significance level are reported based on * p < 0.05, ** p < 0.01, *** p < 0.001

^b^Comparative Fit Index (CFI)

Results from the first models showed that a higher income decreased the likelihood of belonging to the *neutralists* or *hedonists* classes compared to the *maximalists* class (Table 3, models 1). Level of education did not significantly influence class membership. While controlling for confounders (Table 3, models 2), the effect of income decreased but remained significant for both classes compared to those in the *maximalists* class. The effect of education remained insignificant.

When introducing the mediators (Table 3, models 3), the income effect persisted for those in the *neutralists* class but disappeared for those in the *hedonists* class, both compared to those in the *maximalists* class. Instead, those with a higher level of education were significantly more likely to belong to the *hedonists* class compared to the *maximalists* class. Having a future focus was negatively associated with the likelihood of belonging to either the *neutralists* or *hedonists* classes compared to the *maximalists* class, but future focus was not associated with income or educational level. Yet, the influence of having a future focus on class membership was stronger that the effects of the SEP variables on class membership. Financial strain was negatively associated with income but was not associated with class membership. No mediation effects were tested, since neither future focus or financial strain fulfilled the requirement of being associated with both the predictor (SEP) and the outcome (class membership). None of the confounders predicted belonging to the *neutralists* vs. *maximalists* class. Not being in paid employment and being male predicted belonging to the *hedonists* vs. *maximalists* class, the effects of these confounders exceeded the influence of education and income.

## Discussion

This study identified subtle socioeconomic inequalities in the perceived importance of health compared to other life domains, which could not be explained by future focus or financial strain. Based on the importance attached to health (assessed as not being ill and living a long life) and seven other life domains, we identified three latent classes: (1) those in the *neutralists* class considered health and other life domains mostly neutral or unimportant, (2) those in the *hedonists* class considered most life domains important, with the exception of living a long life, work, and religion, and (3) those in the *maximalists* class considered living a long life and not being ill important, as well as most other life domains.

Income inequalities in the importance of health compared to other life domains were identified in nearly all models (a low income increased the likelihood of belonging to the *neutralists* vs. *maximalists* class and thus considering not being ill less important), and educational inequalities were identified in one model (a higher educational level increased the likelihood of belonging to the *hedonists* vs. *maximalists* class and thus considering living a long life less important). To our knowledge, only one previous study by Bowling [12] also assessed a social gradient in the importance of health (though only based on descriptive statistics). Bowling found a trend according to occupational level (those in low occupational classes were somewhat more likely to mention their own health as the most important thing in life than those in the highest occupational class), but identified no consistent trend according to income or education. [12].

Having a lower income and low future focus increased the likelihood of belonging to the *neutralists* vs. *maximalists* class. Participants in the *neutralists* class may have been fully pre-occupied by their challenging circumstances, which may explain their indifferent or somewhat negative stance on the importance of not being ill and various other life domains [38]. This indifference may also function as a coping strategy for dealing with multiple disadvantages [39]. Another explanation could be that people with lower incomes are more frequently ill, which could normalize being ill, and result in a lower perceived importance of not being ill compared to other life domains. However, neither future focus nor financial strain could explain these socioeconomic inequalities in the importance of health and other life domains. Although associated with the importance of health and other life domains, future focus was not linked to the SEP indicators (education nor income) in this sample. The relatively high prevalence of financial strain in all three classes might explain why it was not identified as an explanatory factor for class membership.

In addition to the socioeconomic effects, being in paid employment also had a substantial influence on the importance of health and other life domains. Having no paid or unpaid employment has been associated with many stressors and a depletion of financial and social resources [40]. The associated decrease in social participation [41] combined with the stress of being unemployed could lead to a general feeling of disengagement [40], which could explain the lower importance ratings among those in the *hedonists* class compared to those in the *maximalists* class. The strong influence of employment might also partially capture the effect of health status on the perceived importance of living a long life, as people might be excluded from work due to their health status [26]. Several strengths and limitations should be raised. Most importantly, findings from this study cannot be causally interpreted since cross-sectional data was used. It remains possible that class membership impacted the mediators and SEP in the opposite direction. To be able to draw a causal conclusion about the relationship between SEP and the perceived importance of health compared to other life domains, the findings of this study need to be followed up with a longitudinal study. Moreover, our sample was stratified based on income to ensure a high representation of those with lower incomes, while keeping the sample representative of the Dutch population for education, age, gender, and province. This is a strength because those with lower incomes are often underrepresented, even in research on socioeconomic inequalities. Yet, as a result of the oversampling of low income participants, the effect size of income may have been slightly amplified. We anticipate that a lower income would also be associated with a lower perceived importance of health in the Dutch population. Our survey used a selection of life domains based on Hsieh [11]. This selection may not have reflected important life domains for all participants. Qualitative research into the position of health compared to other life domains and its interpretation may shed more light on this, since explanations for the identified socioeconomic inequalities could also point to socioeconomic differences in reference points or views towards health. Moreover, a reliable statistical method to examine nominal latent outcome variables and multiple mediators has yet to be developed [42]. Therefore, the latent class variable was treated as known, which may have caused downward bias in estimates [43].

Future research could look into other explanations for socioeconomic inequalities in the perceived importance of health. Interesting pathways could include the role of class environment or changes in socioeconomic position or health status. Furthermore, it remains to be studied if socioeconomic inequalities in the perceived importance of health translate into socioeconomic inequalities in health.

## Conclusions

Lower income groups were less likely to consider not being ill important than higher income groups. Those without paid employment and those with a higher educational level were less likely to consider living a long life important than those in lower educational level and those in paid employment. Furthermore, it was noticeable that people with a lower income were less likely to consider any of the included life domains important. Future research should examine if this has detrimental consequences for their (mental) health. Neither future focus nor financial strain contributed to the explanation of the socioeconomic inequalities in the perceived importance of health and other life domains. To support individuals dealing with challenging circumstances in daily life, health-promoting interventions could align to the life domains perceived important to reach their target group and to prevent widening socioeconomic inequalities in health.

## Supplementary Information

**Additional file 1.**

## Data Availability

The datasets used and/or analyzed during the current study are available from the corresponding author on reasonable request.

## Abbreviations

SEP: Socioeconomic position
ISCED: International Standard Classification of Education
LCA: Latent Class Analysis
BIC: Bayesian Information Criterion
AIC: Akaike Information Criterion
SEM: structural equation model
CFI: Comparative Fit Index
