# Supplementary material for: What’s important to you? Socioeconomic inequalities in the perceived importance of health compared to other life domains
Source: BMC Public Health. 2022 Jan 13;22:86. doi: 10.1186/s12889-022-12508-2 (PMC8759269; doi:10.1186/s12889-022-12508-2)
Supplement: Supplementary file 1 — Additional file 1. [file 12889_2022_12508_MOESM1_ESM.docx]

**SUPPLEMENTARY FILE 1:**

Visual representation of all relationships simultaneously estimated in model 3^[[1]](#footnote-1)^.


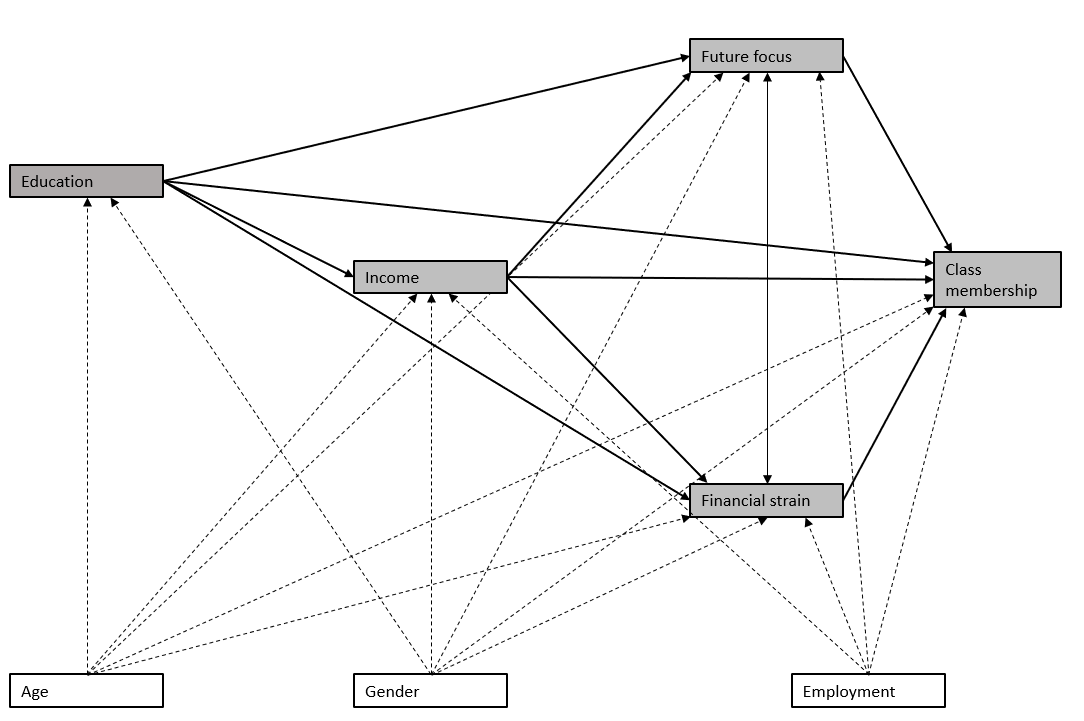


1. The solid bold lines refer to the main relationships tested, the dashed lines refer to confounders adjusted for, and the solid thin line refers to the interaction effect tested between the two mediators. [↑](#footnote-ref-1)
